# Is magnetic resonance imaging (MRI) feasible with an indwelling transpulmonary thermodilution catheter: data from an observational analysis and from a survey

**DOI:** 10.1186/2197-425X-3-S1-A612

**Published:** 2015-10-01

**Authors:** W Huber, A Minning, S Sakka, X Monnet, M Kirov, E Fernández Mondejar, J Wendon, M Maggiorini, C Putensen, J Belda, A Faltlhauser, F Eyer, K Polderman

**Affiliations:** Klinikum rechts der Isar, II. Medizinische Klinik, Munich, Germany; Klinikum Köln-Merheim, Klinik für Anästhesie und operative Intensivmedizin, Köln-Merheim, Germany; Centre Hospitalier Universitaire de Bicètre, Service de Réanimation Médicale, Paris, France; Department of Anesthesiology and Intensive Care Medicine, Northern State Medical University, Arkhangelsk, Russian Federation; Department for Intensive Care, University Hospital Virgen de las Nieves, Granada, Spain; King´s College Hospital, London, United Kingdom; Universitätsspital Zürich, Intensivstation der Abteilung für Innere Medizin, Zurich, Switzerland; Rheinische-Friedrich-Wilhelms-Universität, Bonn, Klinik für Anästhesie und Intensivmedizin, Bonn, Germany; Hospital Clinico Universitario, Valencia, Spain; Kliniken Nordoberpfalz, Medizinische Klinik I, Weiden, Germany; University of Pittsburgh School of Medicine, Neurocritical Care Services, Pittsburgh, United States

## Introduction

Magnetic resonance imaging (MRI) has become a widespread procedure in critical care patients. Some of these patients are equipped with implanted cardiovascular diagnostic or therapeutic devices. The main risks during MRI are movement due to magnetic attraction and burns. The risks depend on quality and quantity of the implanted conductive metallic material as well as on the strength of the magnetic field. There is confusion which patients with which devices can safely undergo MRI. This also applies to transpulmonary thermodilution (TPTD) catheters which currently are not approved to be left in place during MRI. However, risks of delaying MRI until the catheter can be removed, potential harms of removal and risks and costs of repeated arterial cannulation have to be outweighed.

## Objectives

We analyzed a prospectively maintained TPTD database for patients undergoing MRI with PiCCO-TPTD (Pulsiocath; Pulsion Medical Systems SE, Germany) catheters left in place.

## Methods

Patients charts of 16 patients undergoing 20 MRIs with TPTD-catheters were analyzed for side effects. To detect potential catheter dysfunction we compared the last measurements before and after MRI (Wilcoxon-test for paired samples; IBM SPSS 22). Furthermore, a questionnaire regarding the local standard procedure in case of MRI in patients with PiCCO-catheter was sent to 11 experts in hemodynamic monitoring.

## Results

20 MRIs in 16 patients (5 male, 11 female); age 63 ± 7 years; APACHE-II ± 5. No local or systemic complications of the MRI examinations were reported. Hemodynamic measurements before and after MRI were not significant different for any parameter derived from TPTD or pulse contour analysis: CI (4.07 ± 18.35 vs. 4.079 ± 1.41 L/min/²; p = 0.526), GEDVI (775 ± 176 vs. 802 ± 154 ml/m²; p = 0.560), EVLWI (8.7 ± 2.4 vs. 8.9 ± 4.1 ml/kg; p = 0.716), SVRI (1513 ± 472 vs. 1456 ± 527 dyn*s*cm^-5^ *m^-2^; p = 0.550), SVV (13.8 ± 8.2 vs. 12.6 ± 7.8 %; p = 0.146), dPmax (1392 ± 616 vs. 1423 ± 702 mmHg/s; p = 0.881). Questionnaires: Including our center, the catheters were removed in five centers in general and left in situ in six centers. In one center, strategy has changed to removal despite absence of evidence for side effects when not removing the catheter. Seven centers reported on 174 MRI examinations with the TPTD catheter left in situ without any side effect or damage to the catheter. Strategies in the centers removing the catheter comprised delaying the MRI as well as removal of the catheter before MRI with and without replacement after MRI.

## Conclusions

1.) Despite the limitations of a small number of examinations our data do not give hints for harms to the patients or to the TPTD catheter when leaving the TPTD catheter in situ during MRI.

2.) Limited residual risks of the indwelling catheter have to be outweighed to the risks of delayed examination or removal of the catheter and repeated cannulation as well as to the costs of repeated TPTD-catheter Insertion.

